# Single-cell transcriptomic analysis identifies a stress response Schwann cell subtype

**DOI:** 10.1515/med-2025-1186

**Published:** 2025-05-21

**Authors:** Xianfeng Lan, Yanmei Zheng, Yongliang You, Xuejun Wu, Shaojie Wu, Nengfu Chen, Lihong Wang, Wenfu Yang

**Affiliations:** Department of Orthopaedics, Fuzhou Second General Hospital, Fuzhou, 350007, China; The Third Clinical Medical College of Fujian Medical University, Fuzhou, 350007, China; Department of Pharmacy, Fuzhou Second General Hospital, Fuzhou, 350007, China

**Keywords:** ingle-cell RNA sequencing, peripheral nerve injury, stress response, Schwann cell, developmental trajectory

## Abstract

**Background:**

Peripheral nerve injury can lead to sensory, motor, and autonomic nerve dysfunction, significantly impacting patients’ quality of life. Schwann cells (SCs), as key components of the peripheral nervous system, play a crucial role in nerve repair. However, many functionally specialized and flexible SC subtypes remain unidentified. Recent advancements in single-cell transcriptomics have enabled a deeper understanding of SC heterogeneity during peripheral nervous system development.

**Methods:**

In this study, we utilized single-cell transcriptomics to investigate SC heterogeneity in the dorsal root ganglia of both normal and spinal nerve injury mouse models.

**Results:**

We identified a novel SC subtype associated with pressure sensation, which we termed stress response related SCs (SRSCs). These cells are terminally differentiated and highly express the pressure-sensing gene *Npy*. Following peripheral nerve injury, SRSCs function as stimulus receptors, receiving external stimuli and transmitting signals to typical repair SCs via the SPP1 signaling network. This interaction promotes dedifferentiation and facilitates injury repair.

**Conclusion:**

Our findings enhance the understanding of SC heterogeneity and reveal SRSCs as key players in nerve repair. These insights provide potential targets for developing novel therapeutic strategies for peripheral nerve diseases.

## 1 Introduction

Peripheral nerve injury (PNI) is primarily caused by traction injuries, laceration, firearm injuries, compressive injuries, ischemia, and other reasons, leading to temporary or lifelong neurological dysfunction. Its pathological changes include impaired axoplasmic transport, axonal degeneration, Schwann cell (SC) damage, segmental demyelination, and complete Wallerian degeneration [1]. PNI may have several causes, such as trauma and iatrogenic interventions, which can lead to structural loss and/or functional impairment [2]. These changes may result in partial or complete loss of motor and sensory function, physical disability, and neuropathic pain, which in turn can affect the quality of life. According to some studies, up to 1 million people worldwide suffer from PNI each year. The incidence rate in developed countries is about 0.18%, and this proportion is even higher in developing countries [3]. Therefore, peripheral nerve injury is a significant clinical and public health issue.

Unlike the central nervous system, peripheral nerves can effectively regenerate after injury depending on the site and extent of the damage [4]. The ability of peripheral nerves to autonomously regenerate after injury mainly depends on the role of intrinsic supporting cells, such as SCs. As the main glial cells within the peripheral nervous system and the cells that form myelin, SCs play a crucial role in nerve regeneration. After the injury, SCs create favorable conditions for nerve recovery through various mechanisms [5,6,7].

Research shows that SCs quickly dedifferentiate after injury, characterized by losing myelin and reverting to a state similar to early development. They begin to proliferate and migrate to the site of injury, actively participating in the clearance of cellular debris, degenerating axons, and other harmful substances in the damaged area, creating a suitable environment for subsequent repair. In addition, they secrete a large amount of neurotrophic factors, such as nerve growth factor (NGF), brain-derived neurotrophic factor, etc., which can promote the survival of neurons and the regeneration of axons. At the same time, they also secrete extracellular matrix components to help build a scaffold that supports axonal growth. As the axons regenerate, SCs re-envelop the axons and begin the process of remyelination, gradually restoring the normal function and conduction characteristics of the nerve [6,7,8,9,10,11].

Despite the important role that SCs play in the repair of peripheral nervous system injuries, there are still many specific, functionally flexible, and specialized SCs that are not well understood within the entire organism. Recent technological advances in single-cell transcriptomics have revealed the molecular characteristics of SCs during the development of peripheral nerves and the cellular composition of mature nerves, which will help to further unravel the diversity of SC functions and their cellular interactions within the PNS [12,13,14,15].

In this study, we utilized bioinformatics techniques to investigate the cellular states of SC subtypes in the dorsal root ganglion (DRG), identified a novel SC subtype distinct from the typical nerve repair function SCs, which is a stress-responsive SC subtype. We term this as SRSCs and elucidated its mechanism of action, which may provide new therapeutic targets and treatment strategies for nerve injury repair.

## 2 Methods

### 2.1 Quality control and integration for single-cell RNA-seq (scRNA-seq)

The scRNA-seq data analysis was conducted in the R environment (v.4.0.2) using Seurat (v4.3.0) [16]. Quality control measures were applied by filtering out cells with mitochondrial genes >15%, ribosomal genes >15%, and cells that detected <200 or >5,000 genes. We followed standard quality control criteria when evaluating SCs, removing only outliers. The remaining high-quality cells were then processed through normalization, identification of highly variable genes (HVG), data scaling, and principal component analysis (PCA).

PCA was employed as an initial dimensionality reduction step to identify principal components (PCs) that capture the most significant variation in the dataset. These PCs were subsequently used for downstream clustering and batch effect correction. To further enhance visualization and facilitate the interpretation of cellular heterogeneity, we applied the Uniform Manifold Approximation and Projection (UMAP) technique using the RunUMAP function. Unlike PCA, which maintains a linear structure, UMAP provides a nonlinear projection that preserves local and global relationships in the data more effectively.

To mitigate batch effects across the integrated scRNA-seq datasets, we applied the Harmony package [17], following the recommended guidelines. After harmony integration, UMAP was used to generate a two-dimensional representation of the data. The hyperparameters for UMAP were set as follows: n_neighbors = [insert value] and min_dist = [insert value], optimized to balance local and global structure preservation. Clustering was performed using the FindNeighbors and FindClusters functions, with a resolution parameter set to 0.4. Throughout the analysis, default settings were used for standard procedures unless otherwise specified.

By utilizing both PCA and UMAP, we aimed to leverage the strengths of each method – PCA for feature extraction and dimensionality reduction in the initial processing stages and UMAP for intuitive and high-resolution visualization of cellular heterogeneity.

### 2.2 Cell annotation, differentially expressed genes (DEGs), and marker genes identification

The cell type annotation was performed using the Single R package, which is capable of associating gene expression of different cell types with single-cell resolution cell gene expression. By utilizing the expression of HVGs, the Single R package calculates the correlation between the gene expression of single-cell samples and the gene expression of cell types in the reference database. By iteratively eliminating the weakest correlation of each cell type, the corresponding cell type can be identified [18]. In this study, we annotated the samples using the “ref_Human_all” database within the Single R package. Subsequently, we used the “FindAllMarkers” function in the Seurat package to identify DEGs for further analysis, applying a threshold of |log2FoldChange| > 2 and an adjusted *P*-value < 0.05. Finally, we utilized the “FindMarkers” function in the Seurat package to identify cell type-specific marker genes.

### 2.3 GO enrichment analysis

In the annotation analysis, the GO functional enrichment analysis was performed using the David database and the Metascape database. The results were visualized using the ggplot2 package [19,20].

### 2.4 Cell–cell communication analysis

Cellular communication, driven by the interactions of ligands and receptors on the cell surface, is essential for a multitude of biological processes. To explore the crosstalk between various cell types, we employed CellChat 1.5.0 for dissecting the intercellular dialogue. This R package is tailored for deducing and quantifying the communication networks from single-cell RNA sequencing data. By mapping out the interactions of ligands, receptors, and their associated molecules, CellChat models the signaling pathways between cells. It leverages the expression patterns of these molecules across different cell types to deduce their mutual interactions and to identify the enriched interactions between receptors and ligands in the context of two interacting cell populations [21].

### 2.5 Cellular trajectory analysis (pseudotime analysis)

Cells are in a constant state of dynamic change, transitioning from one cell type to another, which leads to alterations in gene expression and functional states [22]. Pseudotime analysis arranges each cell along the corresponding cellular trajectory, representing a pseudotemporal sequence, and groups cells into different differentiation states by employing a curve of gene expression. We conducted pseudotime analysis on SC subtypes using the R packages Monocle 2 and CytoTRACE [23]. To explore the differentiation trajectories and associated genes of different states of SC subtypes, we used CytoTRACE to calculate the pseudodifferentiation frequency of each cell to infer its differentiation state and employed the “plot_cell_trajectory” function to order cells along the pseudotime. The “BEAM” function was utilized to identify genes responsible for cellular branching and differentiation.

### 2.6 Immunofluorescence staining

Mice at defined ages were anesthetized before sacrifice and then perfused with ice-cold phosphate-buffered saline (PBS) followed by 2% paraformaldehyde. The L4 and L5 DRGs were dissected, fixed in 2% paraformaldehyde for 10 min, dehydrated in 25% sucrose at 4°C overnight, embedded in OCT, and processed for cryo-sections at 12 μm.

For immunohistochemistry, cryo-sections were permeabilized and blocked in blocking buffer (0.4% Triton X-100 and 3% normal BSA in PBS) for 1 h at room temperature (RT) and overlaid with primary GAL antibody (Invitrogen, PA5-25649) and primary NPY antibody (Invitrogen, ABS 028-08-02) overnight at 4°C. After washing with PBS, sections were incubated with secondary antibodies conjugated to Cy2, Cy3, or Cy5 (1:1,000) for 1 h at RT, stained with 4′,6-diamidino-2-phenylindole for 10 min, washed three times in PBS and then mounted in Mounting Medium.

### 2.7 Ligand–receptor interaction analysis

To identify significant ligand–receptor interactions, we utilized CellPhoneDB (or other relevant tools, if applicable) to analyze cell–cell communication based on single-cell transcriptomic data. The statistical significance of ligand–receptor interactions was assessed using permutation tests. Specifically, we performed 1,000 permutations by randomly shuffling cell-type labels to generate a null distribution for each interaction. Interactions were considered statistically significant if their *p*-value was ≤0.05 after multiple testing correction using the Benjamini–Hochberg (BH) method to control the false discovery rate (FDR). Additionally, we applied a hypergeometric test to determine whether observed ligand–receptor interactions were enriched compared to random expectations. The enrichment score and *p*-values were computed based on the overlap between observed interactions and a predefined ligand–receptor database. Only ligand–receptor pairs with an adjusted *p*-value (FDR) ≤0.05 were retained for further analysis.

### 2.8 Statistical analysis

To identify differentially expressed genes in bulk scRNA-seq, the expression data were analyzed using Seurat. Genes with a Log2 fold change (FC) of 1 or greater and a *p*-value of 0.05 or less were classified as differentially expressed. As depicted in the figure, the data are presented as mean ± standard deviation (SD) or mean ± standard error of the mean (SEM), depending on the context. SD was used to reflect the variability of individual data points, while SEM was used to show the precision of the sample mean. A *p*-value less than 0.05 at the 95% confidence level is considered statistically significant.


**Ethics approval and consent to participate:** Ethical approval for these procedures has been obtained from the Medical Ethics Committee of Fuzhou Second General Hospital (202201167, Fuzhou, China). All methods are reported in accordance with ARRIVE guidelines. The full experimental procedures were carried out under the guidance of the Institutional Animal Care and Use Committee of Fuzhou Second General Hospital (202201167, Fuzhou, China). All Mice were housed in the animal facility of Fuzhou Second General Hospital and were kept pathogen free. Animal Care and Use Committee reviewed and approved the procedures involving the care and use of animals.

## 3 Results

### 3.1 Unbiased clustering identified known cell populations in mice DRG

**Figure 1:**
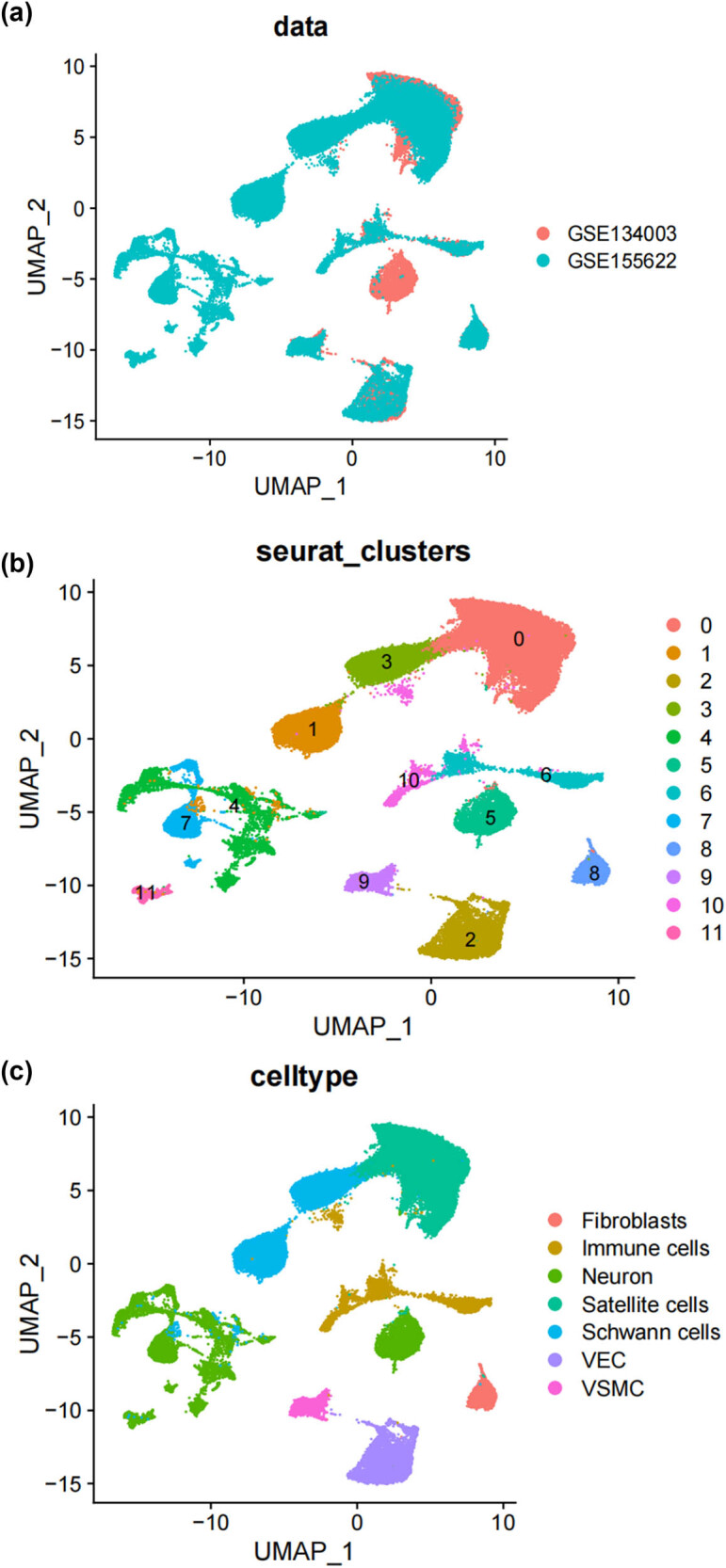

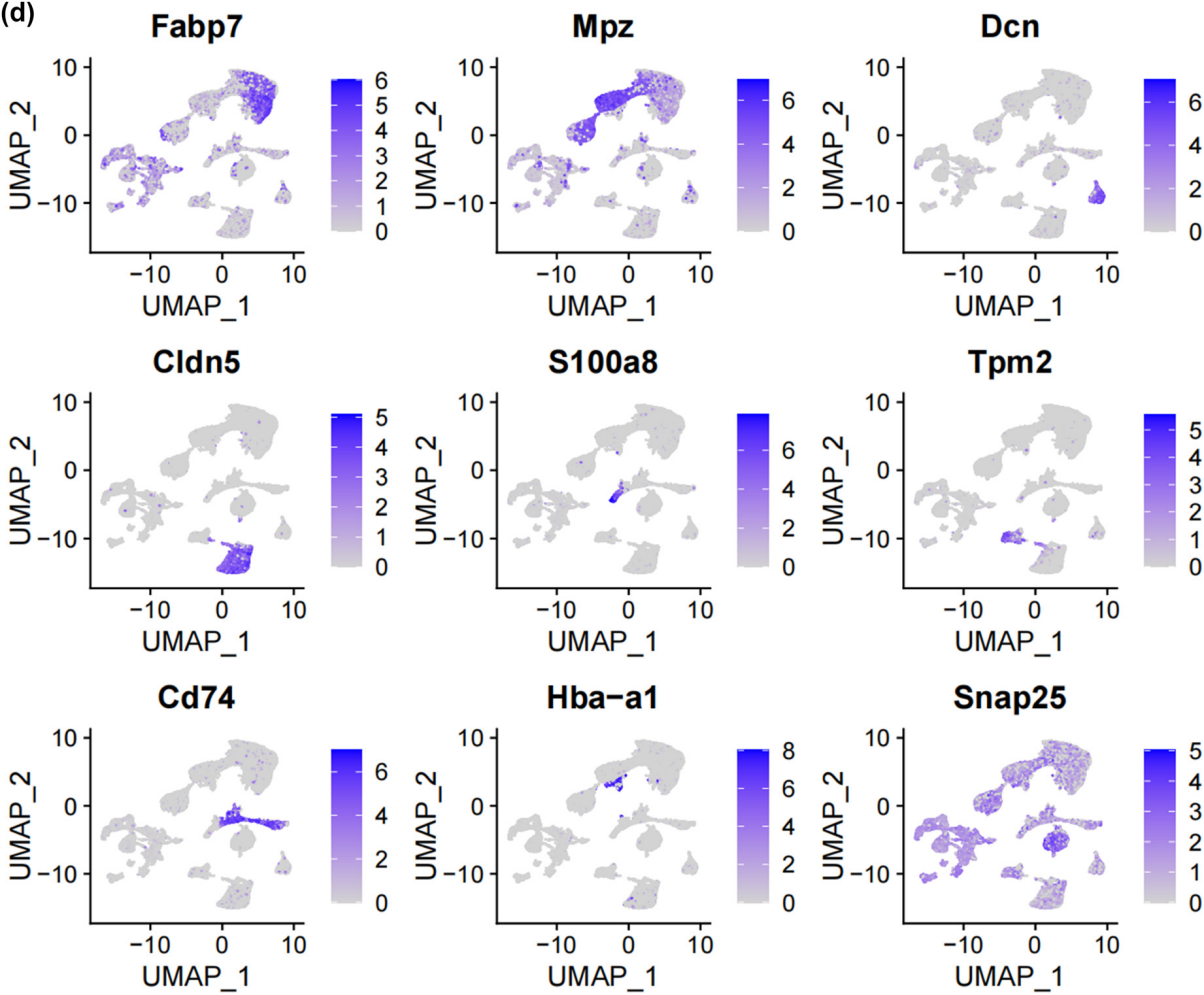
Unbiased clustering identified known cell populations in mice DRG. (a) UMAP plot showing the distribution of each dataset after integrating datasets using the harmony algorithm; (b) and (c) UMAP plot revealing the integrated cell map, with 12 cell clusters (b) of 7 annotated cell types (c). Each dot presents one single cell colored by clusters; (d) UMAP plot showing the scaled expression of representative marker genes across cell types. 8525.

In this study, we analyzed two publicly accessible microarray datasets (GSE134003 [24] and GSE155622 [25]) retrieved from the NCBI Gene Expression Omnibus database (GEO) (https://www.ncbi.nlm.nih.gov/geo/). It includes DRG tissues from 11 normal mice and 14 Spared nerve injury (SNI) mice. Figure 1a displays the single-cell transcriptomic profiles of different datasets, with cluster analysis dividing the cells into 12 clusters, as shown in Figure 1b. Unbiased clustering of the cells identified 7 clusters based on UMAP analyses. Each cluster was annotated based on the top principals, and the marker genes were calculated (Figure 1c and d). In particular, they were as follows: (1) fibroblasts, (2) immune cells, (3) neurons, (4) satellite cells, (5) SCs, (6) vascular endothelial cells, and (10) vascular smooth muscle cell cluster. The profiles of the expression differences of the representative marker genes in the cell populations were demonstrated by statistical quantification to match the biological annotation.

### 3.2 Subtypes of SCs in the DRG

**Figure 2:**
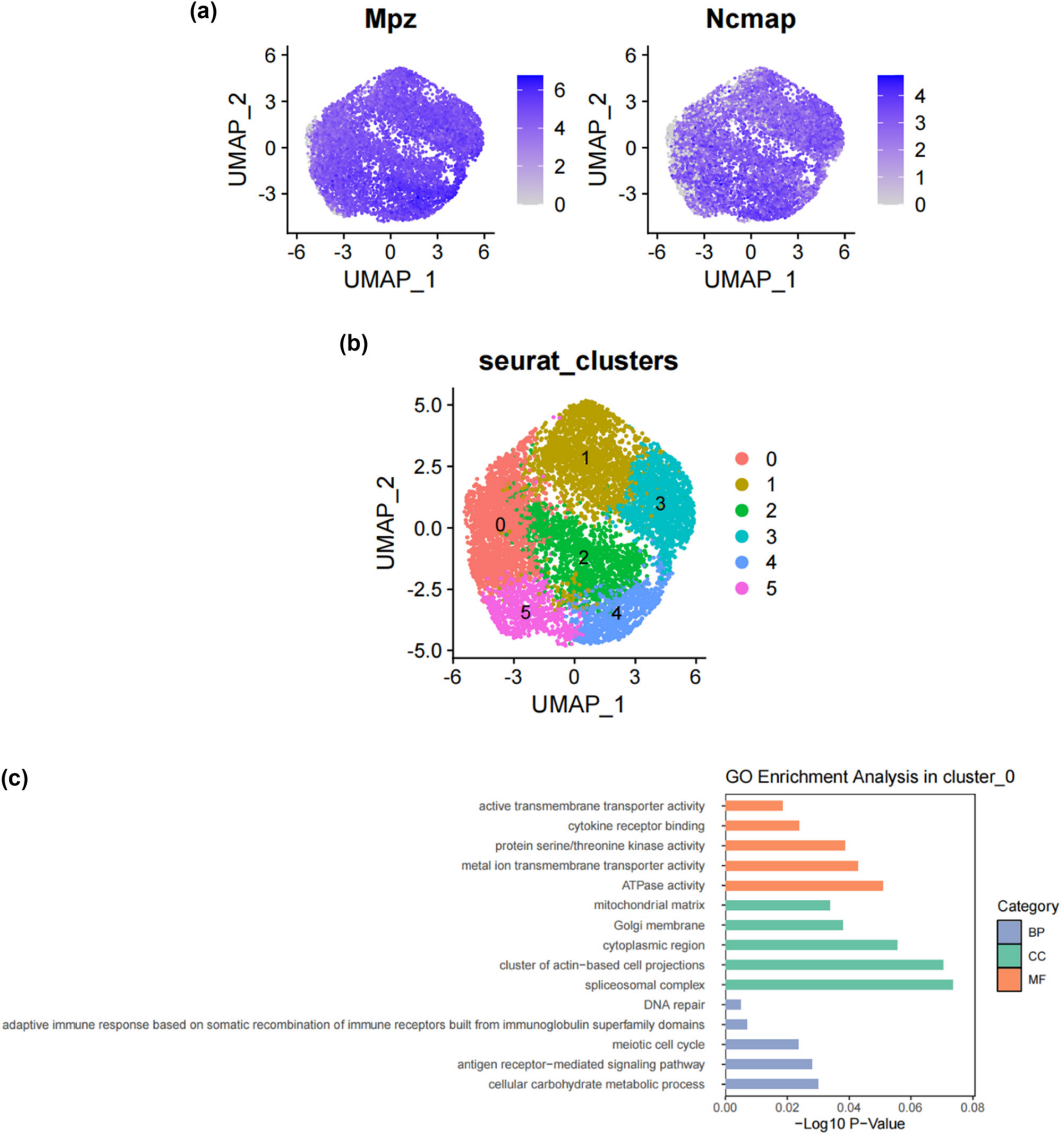

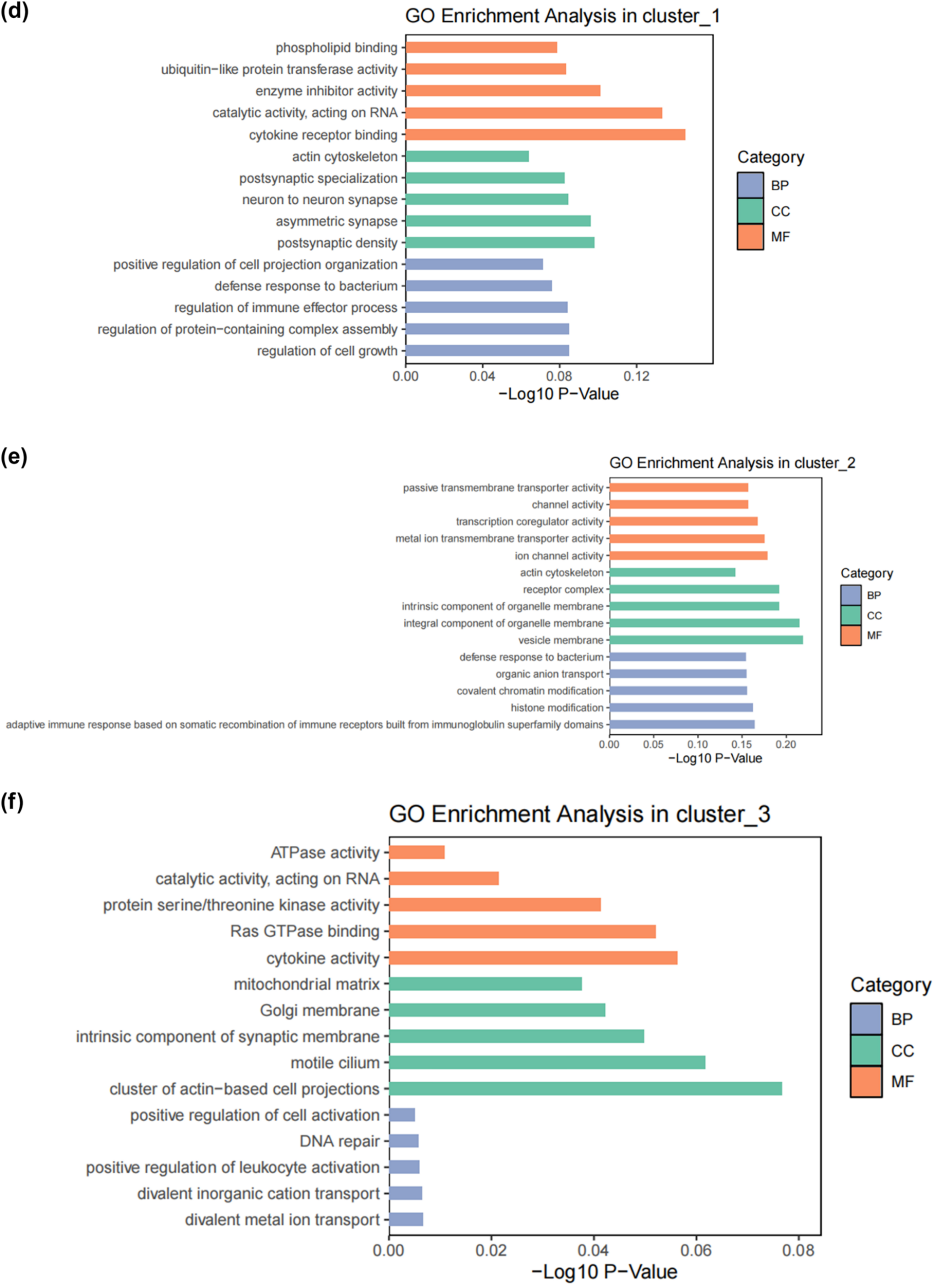

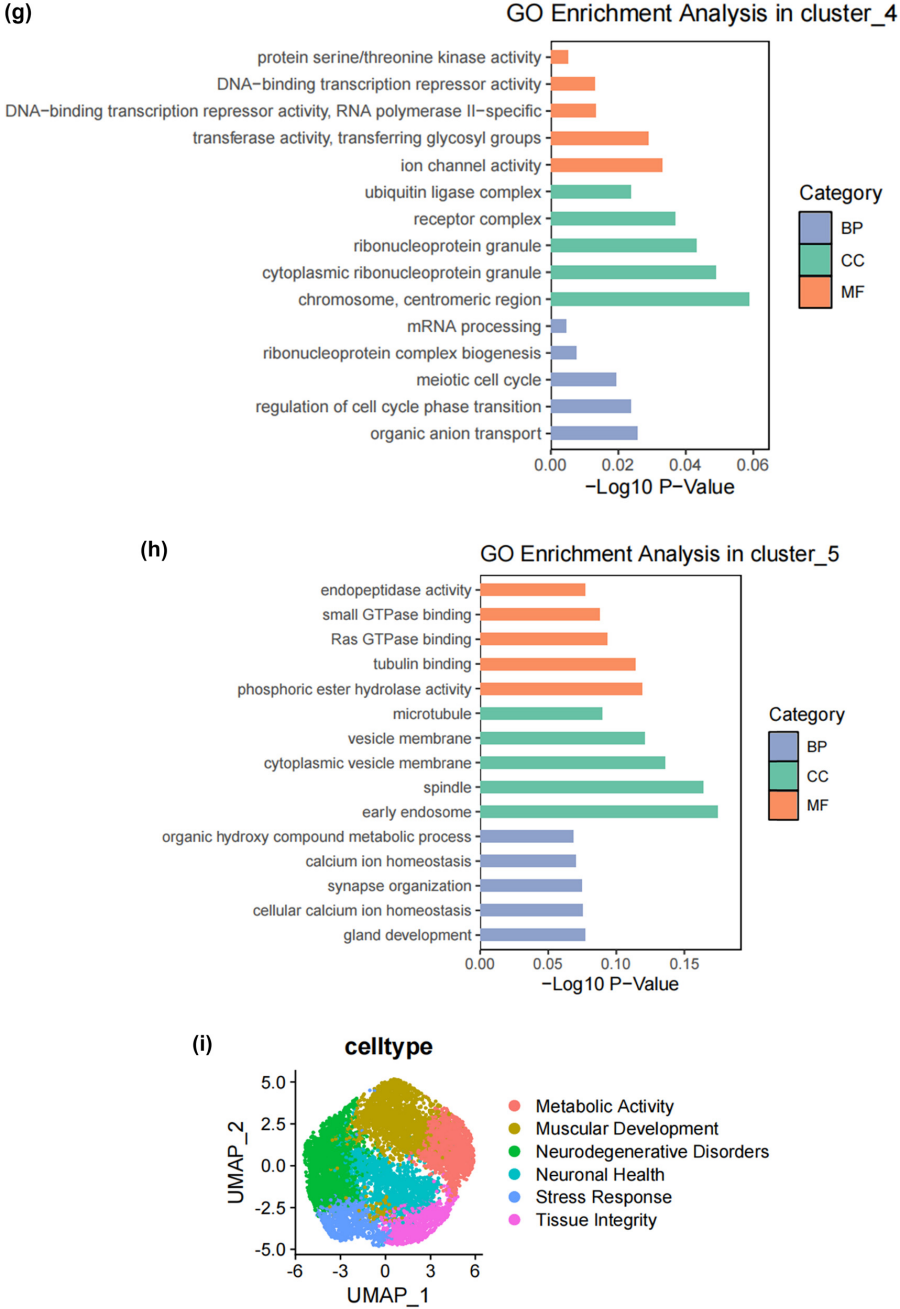

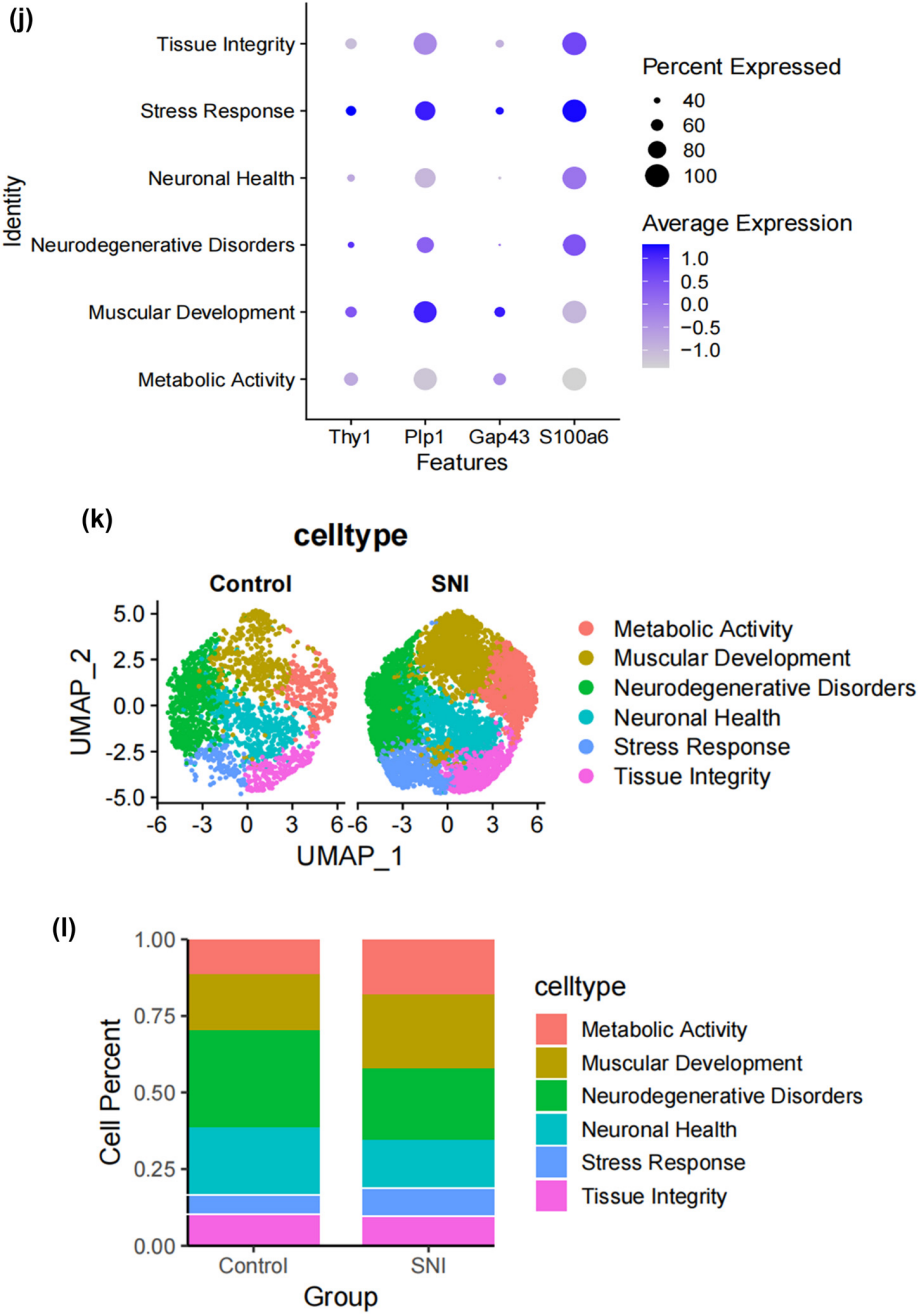
Subtypes of SCs in the DRG. (a) UMAP plot showing representative genes in SCs; (b) UMAP plot of 8525 SCs clustered by annotated cell types; (c)–(h) GO enrichment analysis of six SC subtypes marker genes, shown in terms of biological process (BP), cellular component (CC), and molecular function (MF), BP refers to the biological processes the genes are involved in, CC denotes the cellular components where the genes are localized, and MF describes the molecular functions of the encoded proteins.; (i) UMAP plot showing the named SCs along with GO enrichment analysis; (j) UMAP plot showing the expression levels of selected genes in six cell subsets. UMAP plot (k) and Bar plot (l) showing the cell percentage of each SC subtype in Control and SNI mice.

SCs, as the main glial cells of the peripheral nervous system (PNI), provide structural and nutritional support for axons and supply energy metabolites to neurons, playing a key role in the peripheral nervous system [26]. Subsequently, we extracted SCs from the single-cell map we previously constructed and performed more detailed sub-clustering and annotation on them. Figure 2a shows the extracted SCs highly expressing their surface markers *Mpz* and Ncam [27,28,29,30,31], and Figure 2b demonstrates that SCs are divided into six clusters. Following that, we conducted GO analysis to understand the molecular functions of the related clusters and discovered functional differences among each sub-cluster. Cluster 0 is mainly involved in biological processes such as cellular carbohydrate metabolism and the activity of transmembrane transport proteins; Cluster 1 is mainly involved in biological processes such as the formation of the actin cytoskeleton, metal ion transmembrane transport, cell growth movement, and signal transduction; Cluster 2 is mainly involved in biological processes such as adaptive immune response, maintenance of cellular homeostasis, and transcription coactivator activity; Cluster 3 is mainly involved in biological processes such as catalytic enzyme activity, cellular respiration and energy production, and maintenance of protrusive structures; Cluster 4 is mainly involved in biological processes such as cell signal reception and transmission, regulation of cell cycle phase transition, and cell division; Cluster 5 is mainly involved in biological processes such as gland development and maturation, and the assembly of synapses (Figure 2c–h). Based on their distinct molecular biological functions, we have named these six clusters as follows (Figure 2i): metabolic activity-related SCs (MASCs), muscular development-related SCs (MDSCs), neurodegenerative disorders-related SCs (NDSCs), neuronal health-related SCs (NHSCs), stress response related SCs (SRSCs), and tissue integrity related SCs (TISCs). Surprisingly, we found that the gene *Thy1*, which is involved in cell-to-cell interactions in the brain, the myelin-forming gene *Plp1*, the key component of the effective nerve regeneration response *Gap43*, and the gene *S100a6* that regulates cell cycle progression and differentiation, are highly expressed in the SRSC group (Figure 2j and Table A1). Additionally, our analysis revealed a notable increase in both the absolute number and relative proportion of SRSCs in the SNI group compared to the normal group (Figure 2k). This increase suggests that SRSCs may be actively involved in the cellular response to nerve injury. The expansion of this SC subtype indicates a potential functional shift following injury, where SRSCs may play a role in mediating stress response, repair processes, or neuroprotection. To further validate these findings, statistical analysis confirmed that the observed increase in SRSCs was significant, reinforcing their potential importance in the regeneration and recovery of injured peripheral nerves (Figure 2l). With the support of transcriptomic pathway analysis, we further explored whether the observed SC subtypes might represent distinct metabolic states. The differential expression of genes involved in metabolic pathways, such as glycolysis and oxidative phosphorylation, across SC subtypes, suggests that they could be in different metabolic states. This metabolic heterogeneity may reflect the cells’ readiness to adapt to the bioenergetic demands during nerve repair and regeneration processes. After peripheral nerve injury, a series of reactions occur locally, such as oxidative stress, excessive inflammation, ischemia, and insufficient energy supply, leading to a deficiency in chemotactic NGFs and the production of a large number of factors that inhibit nerve regeneration, making nerve regeneration difficult [32,33,34]. SCs, as an important component of the peripheral nervous system, have good plasticity. After nerve injury, they undergo a series of stress responses and re-engage in the formation of myelin, restoring the insulation and signal transmission functions of the nerve. This may also be the reason why the increase in SRSCs in the SNI group. A large number of studies have shown that reducing oxidative stress in a mouse model of acute peripheral nerve injury can improve functional recovery [34,35,36]. Therefore, SRSCs may become a potential target for future treatment strategies for peripheral nerve injury. Furthermore, we have observed a reduction in the ratio of TISCs, which could be due to incomplete repair resulting in pain and the long-term loss of function that ultimately impairs the integrity of the tissue.

### 3.3 The states of SCs in the DRG

**Figure 3:**
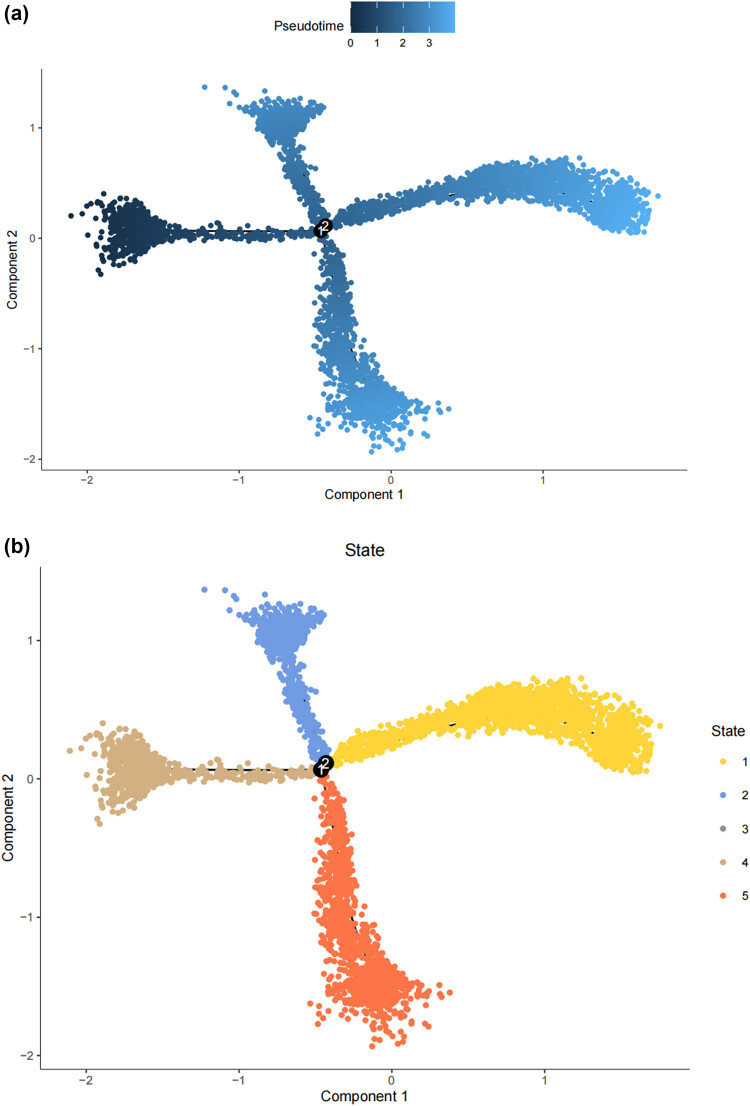

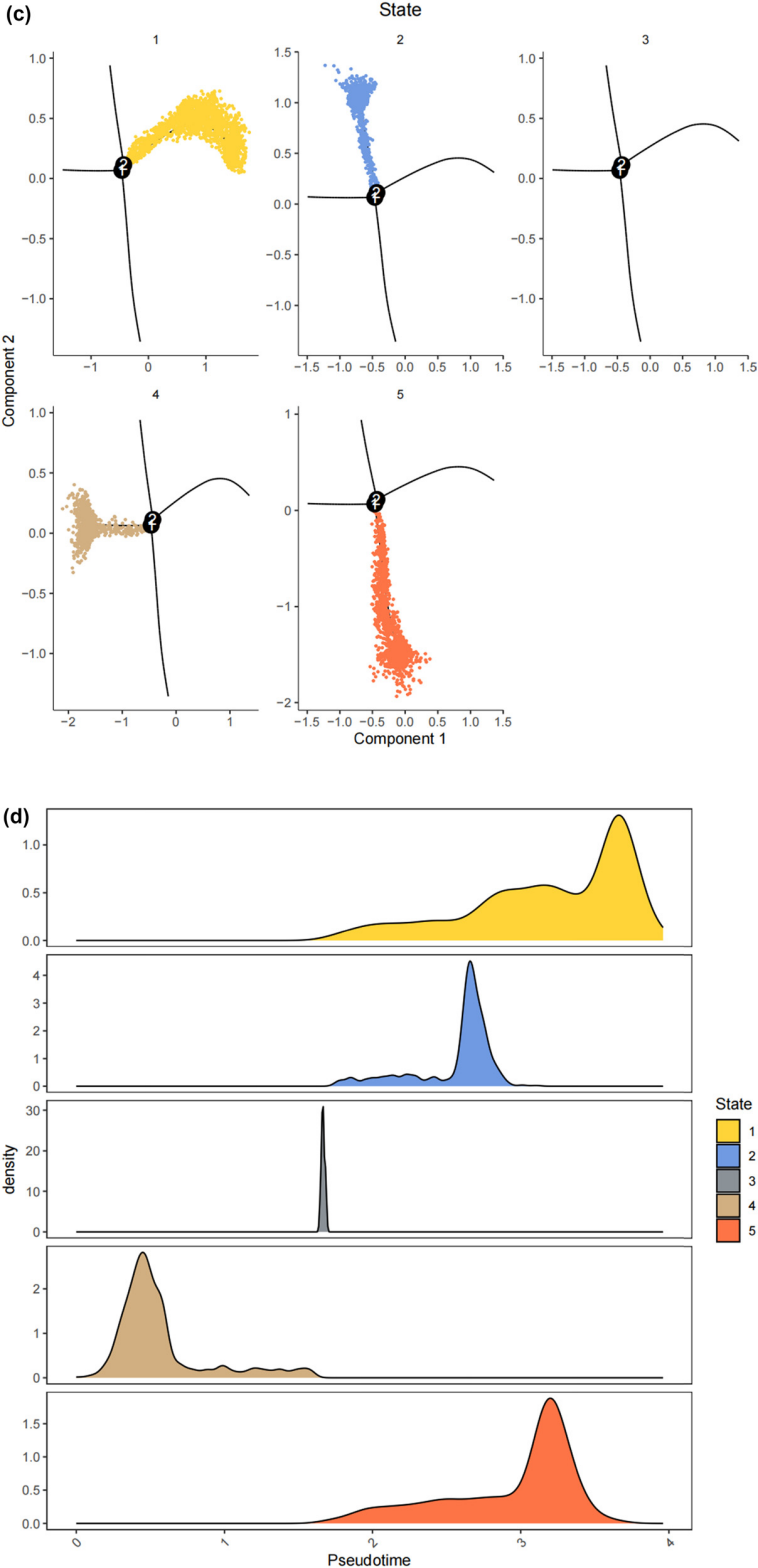

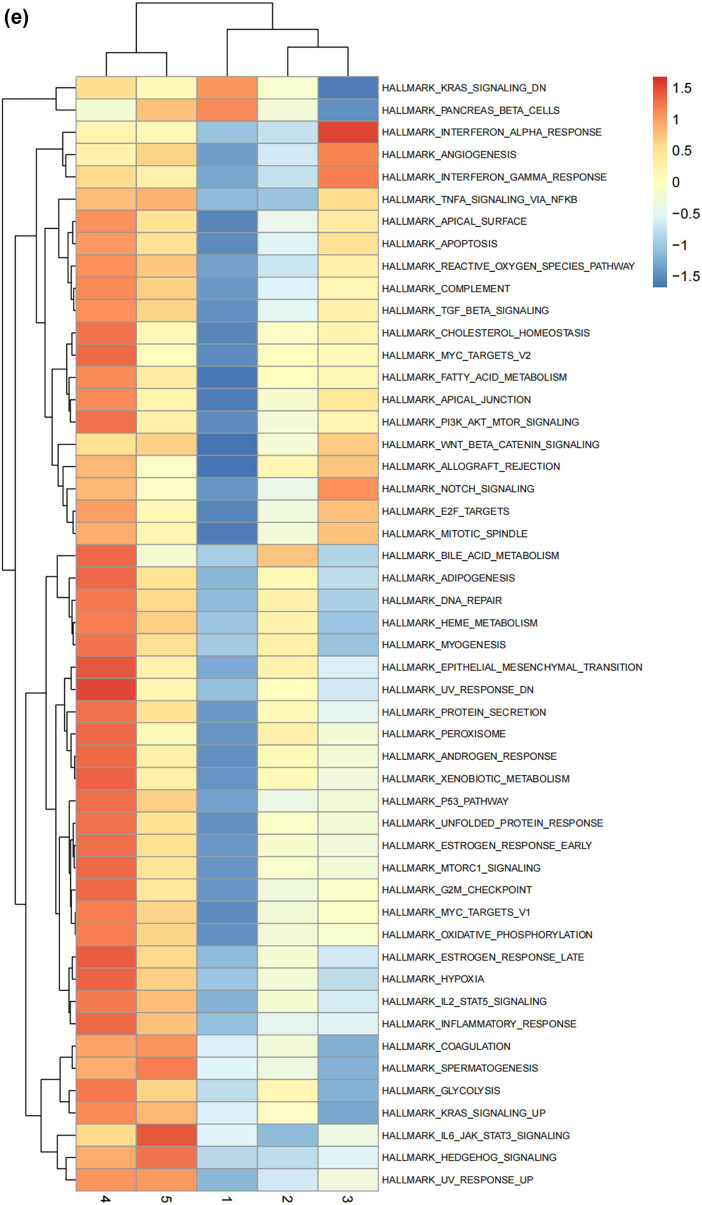
The states of SCs in the DRG. (a) Trajectory plot illustrating the evolutionary trajectory of SCs; (b) trajectory plot illustrating the evolutionary trajectory of SCs colored by cell states; (c) branched heatmap showing genes with highly significant branch-specific expression patterns in the pseudotime trajectory; (d) ridge plot showing the Cell differentiation process of the five states of SCs; and (e) GSVA enrichment analysis of hallmark gene sets in SC subtypes.

Lineage tracing and trajectory inference suggest that the process of cell differentiation may not be synchronized; under the same static conditions, cells at different developmental stages can be observed. Therefore, by analyzing the differentiation trajectory of SCs in the DRG to predict the cellular fate of SCs. After mapping SCs onto the differentiation pathway, bifurcating trajectories were observed (Figure 3a). Pseudo-time trajectories divide SCs into a total of five cellular states, where state 4 SCs differentiate into state 1, 2, 3, or state 5 SCs (Figure 3b and c), and state 1 SCs are at the terminal phase of differentiation (Figure 3d). Gene set variation analysis (GSVA) performed functional analysis on the transcriptomes of single cells in different states to annotate their unique molecular characteristics and biological involvement (Figure 3e). GSVA indicates that state 1 SCs, which are at the end of differentiation, contain many significantly downregulated gene sets, especially MYC_TARGETS_V2 (genes regulated by MYC), FATTY_ACID_METABOLISM (genes involved in fatty acid metabolism), WNT_BETA_CATENIN_SIGNALING (genes involved in cell differentiation and development), ALLOGRAFT_REJECTION (genes involved in immune rejection), and E2F.

### 3.4 Prediction of the differentiation of SC subtypes

**Figure 4:**
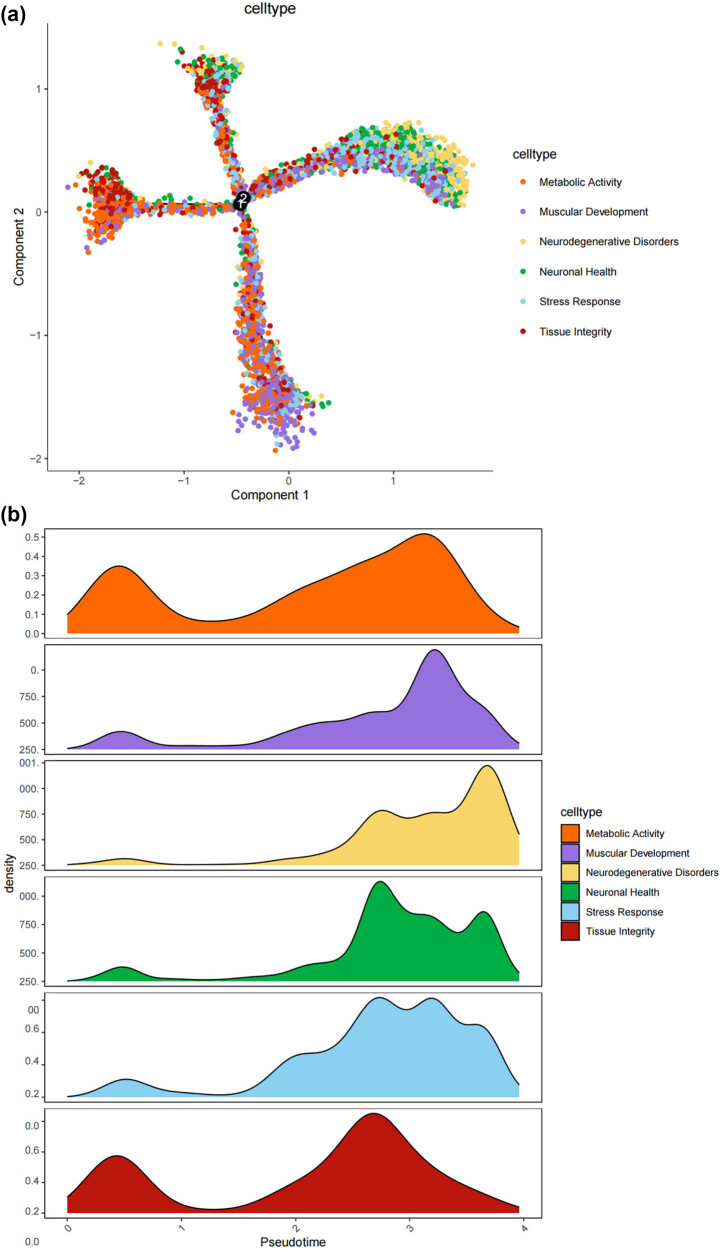

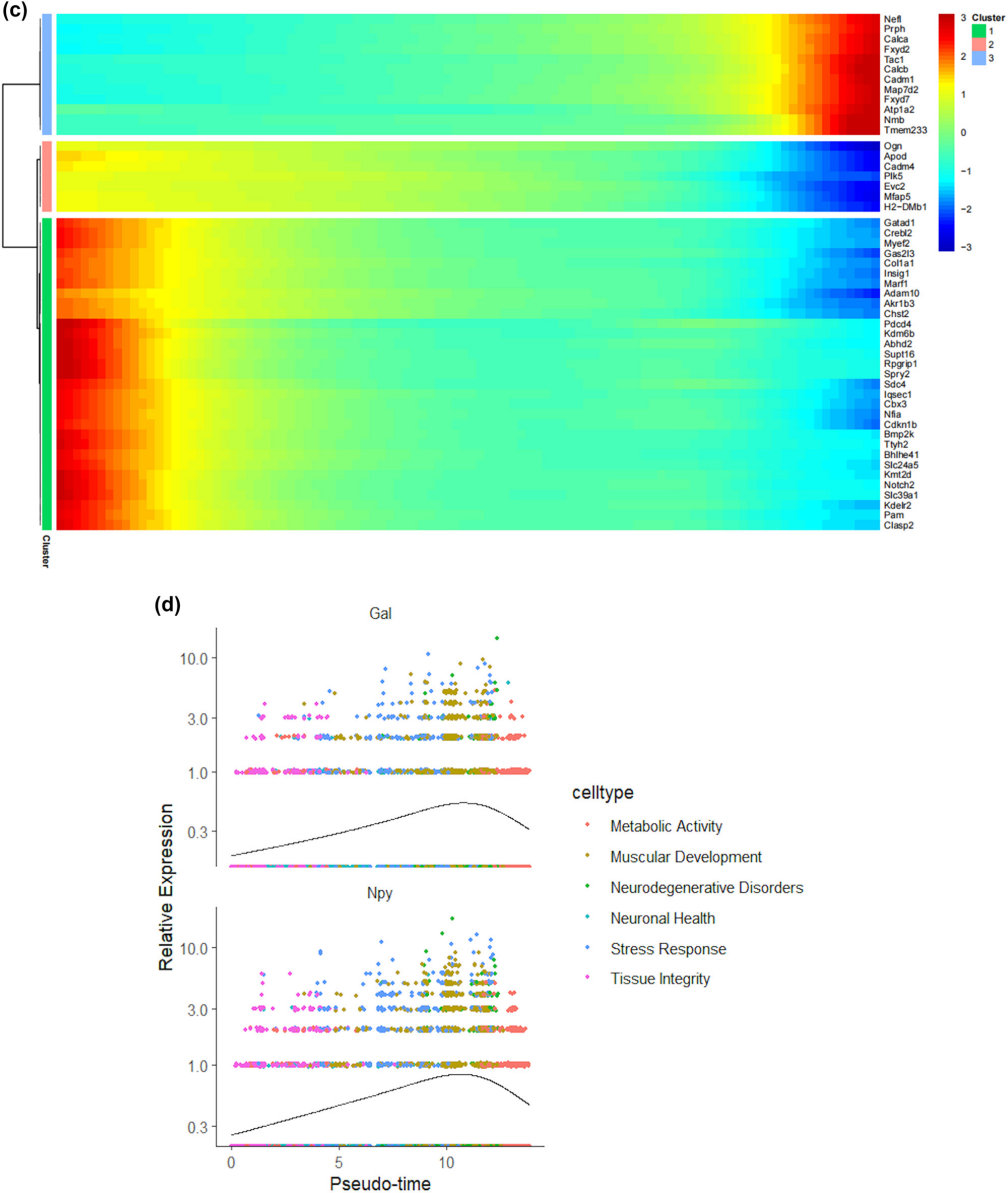

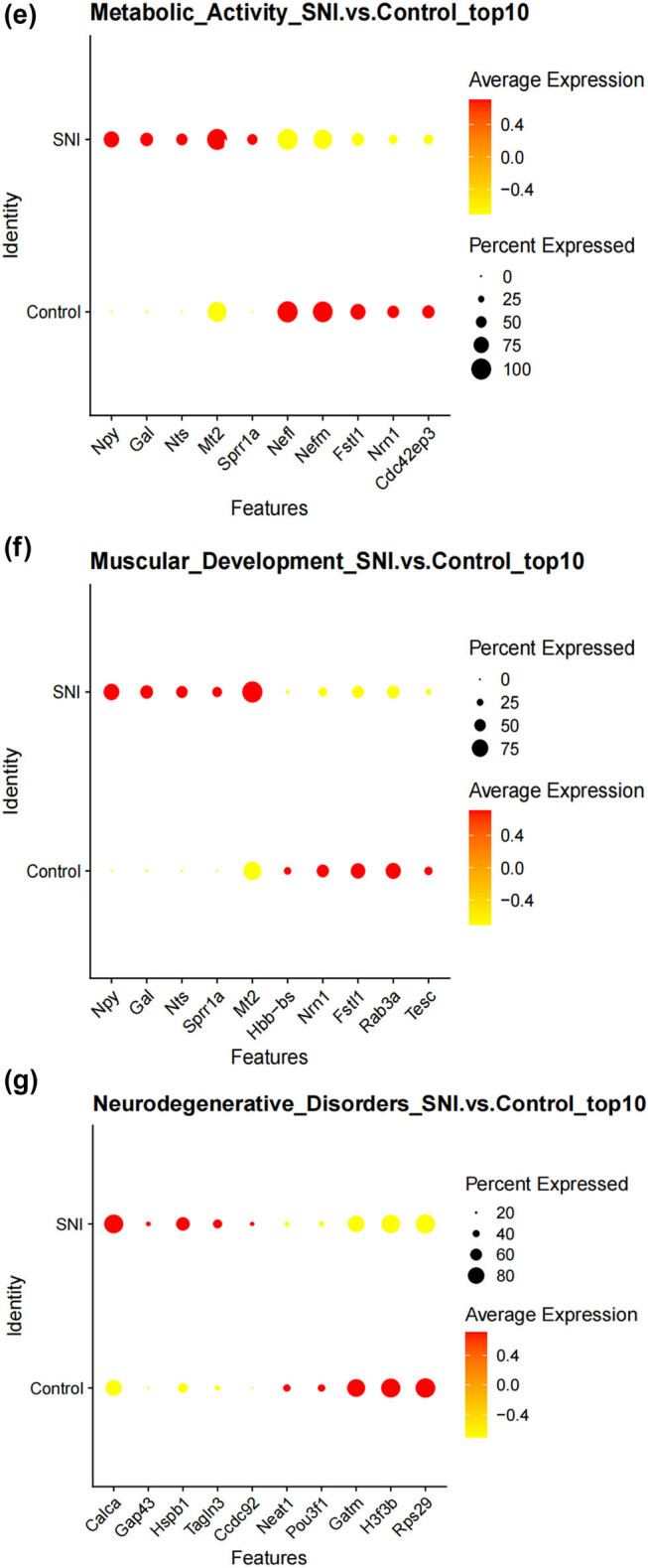

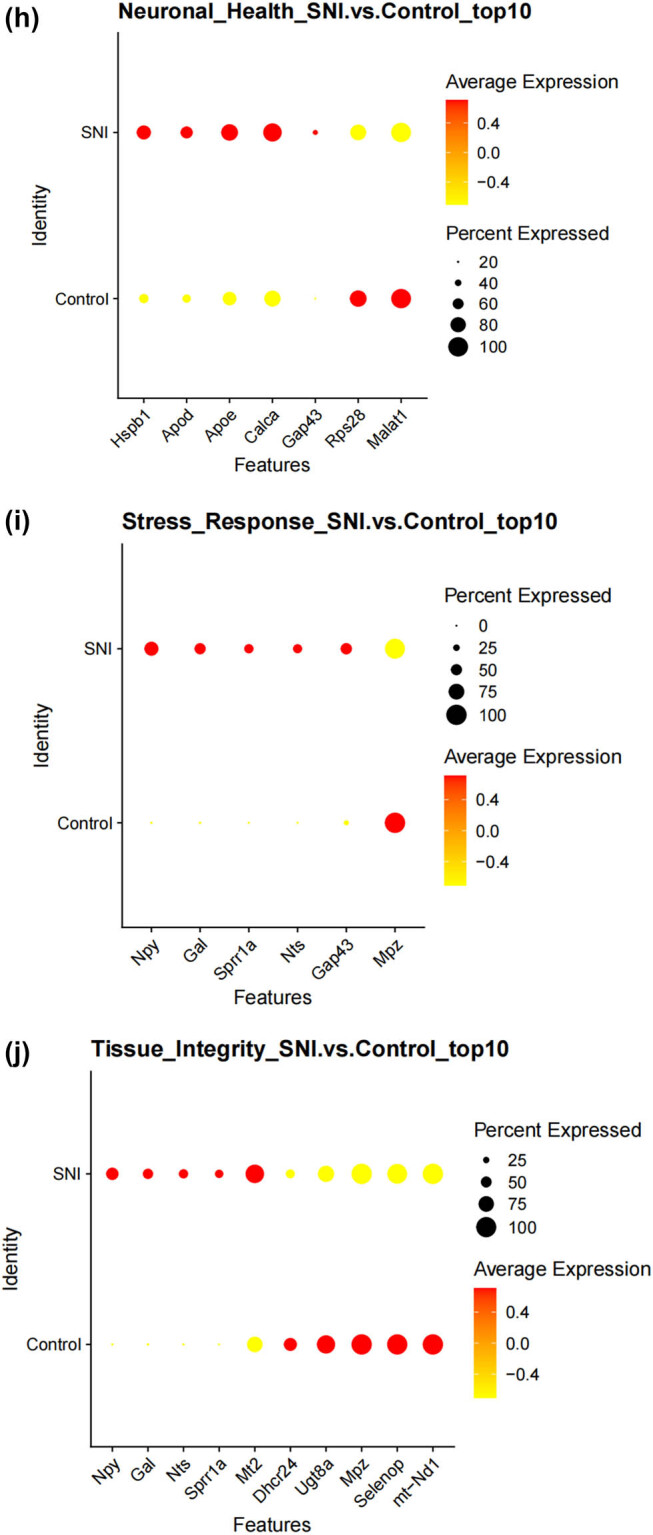

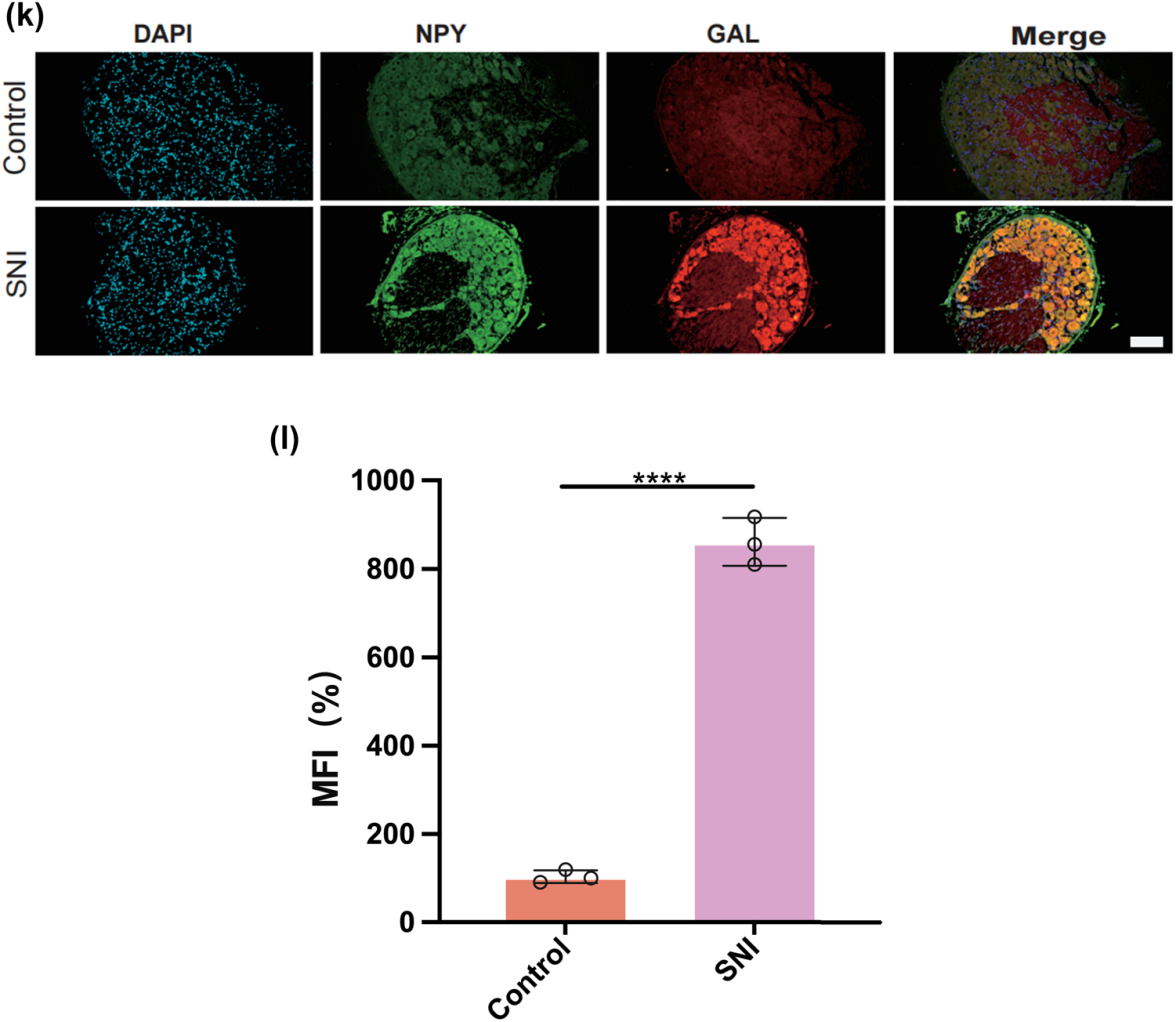
Prediction of the differentiation of SC subtypes. (a) Monocle pseudotime analysis revealing the progression of six SC subtypes; (b) ridge plot of six SC subtypes; (c) heatmap showing the scaled expression of differently expressed genes in three clusters as in (b); (d) relative gene expression levels of *Gal* and *Npy* across different cell types.; (e)–(j) dot plot revealing the top 10 marker genes of indicated SC subtypes in control and SNI mice; (k) immunofluorescence staining of NPY and GAL. Scale bar = 20 μm. (l) Quantification of K.

We thus determined the pseudo-time sequence of SC subtypes. Throughout the cell’s entire hierarchical life process, various factors stimulate changes in gene expression, transforming them into different cell subtypes or states. Gaining a deeper understanding of the functional changes in SCs is crucial for our knowledge of the growth and development of the peripheral nervous system and the disease process. Subsequently, we inferred the differentiation process and potential of different SC subpopulations from a single-cell level. As shown in Figure 4a and b, MASCs and TISCs appear to be concentrated in state 4 SCs, with state 1 primarily composed of NDSCs. It can be seen that MASCs and TISCs, which have high metabolic activity and are in a proliferative state, have the lowest degree of differentiation and have the potential to differentiate into other cell types. They may be the progenitor cells in the developmental trajectory of SCs and gradually develop into MDSCs, NDSCs, NHSCs, SRSCs, and TISCs, while NDSCs, being at the terminal stage, do not have the potential for further development. The heatmap illustrates the changes in gene expression along the pseudo-time trajectory branches. These genes are clustered into three modules with distinct expression patterns, where different colors represent the levels of gene expression. We can observe that cluster1 (NDSCs) exhibits low expression of genes involved in the regulation of cell proliferation, metabolism, cell cycle, cell signaling, and cell death, such as GATAD1, GAS2L3, ADAM10, SDC4, LQSEC1, CBX3, NFIA, CDKNLB, etc.; however, cluster3 (SRSCs) shows high expression of genes that maintain cell structure, signaling, metabolic regulation, and cell–cell interactions, for example, NEFL, PRPH, CALCA, FXYD2, TACL, CALCB, CADM1, MAP7D2, FXYD7, ATP1A2, NMB, TMEM233 (Figure 4c). In particular, the SRSCs, characterized by their unique gene expression signature, are hypothesized to play a pivotal role in the initial stress response to nerve injury. The upregulation of stress-related genes in SRSCs, such as *Npy* and *Gal,* suggests their active involvement in modulating cellular responses to stress and facilitating subsequent repair mechanisms. This distinct gene expression profile not only highlights the potential role of SRSCs in the immediate stress response but also indicates their importance in the subsequent repair and regeneration phases following nerve injury (Figure 4d). Next, we screened for differentially expressed genes in these six subtypes, and Figure 4e–j display the distribution of the top 10 differentially expressed genes. After SNI occurs, the antioxidant *Mt2* that maintains the homeostasis of intracellular metal ions and detoxifies heavy metals is highly expressed in MASCs, MDSCs, and TISCs, which is consistent with the results of the previous pseudo-time sequencing. It is worth noting that the stress response genes *Npy* and *Gal* are highly expressed in SRSCs, indicating that after SNI, various SC subtypes perform their respective duties. MASCs, MDSCs, and TISCs exert the typical functions of SCs, that is, to repair damaged nerves in an inflammatory environment, while SRSCs sense the stimuli. Next, we explored the expression level of NPY and GAL in the DRG of normal and SNI mice. The results of the immunofluorescence showed that the expression of both was significantly increased in the SNI group, suggesting that after peripheral nerve injury, there is a noticeable stress response in the DRG (Figure 4k and l). We observed an increase in NPY expression within SCs following spinal nerve injury (SNI). Given that NPY is typically stored in dense-core vesicles of neurons and may also be present in non-neuronal cells, the observed increase in NPY levels raises the question of whether SNI merely alters the storage of NPY within vesicles or whether it globally increases the number of dense-core vesicles. Although our data suggest that SRSCs may be the source of elevated NPY expression post-injury, we also acknowledge the possibility that this increase could be attributed to neurons ensheathed by SCs. To address this issue, future studies might involve detailed ultrastructural analysis to assess the density and distribution of dense-core vesicles in SCs before and after SNI. However, based on our current data, we hypothesize that the increase in NPY expression is more likely associated with a functional response of SCs to injury rather than a simple quantitative change in vesicle numbers.

### 3.5 SPP1 signaling induces distinct SC interactions in DRG

**Figure 5:**
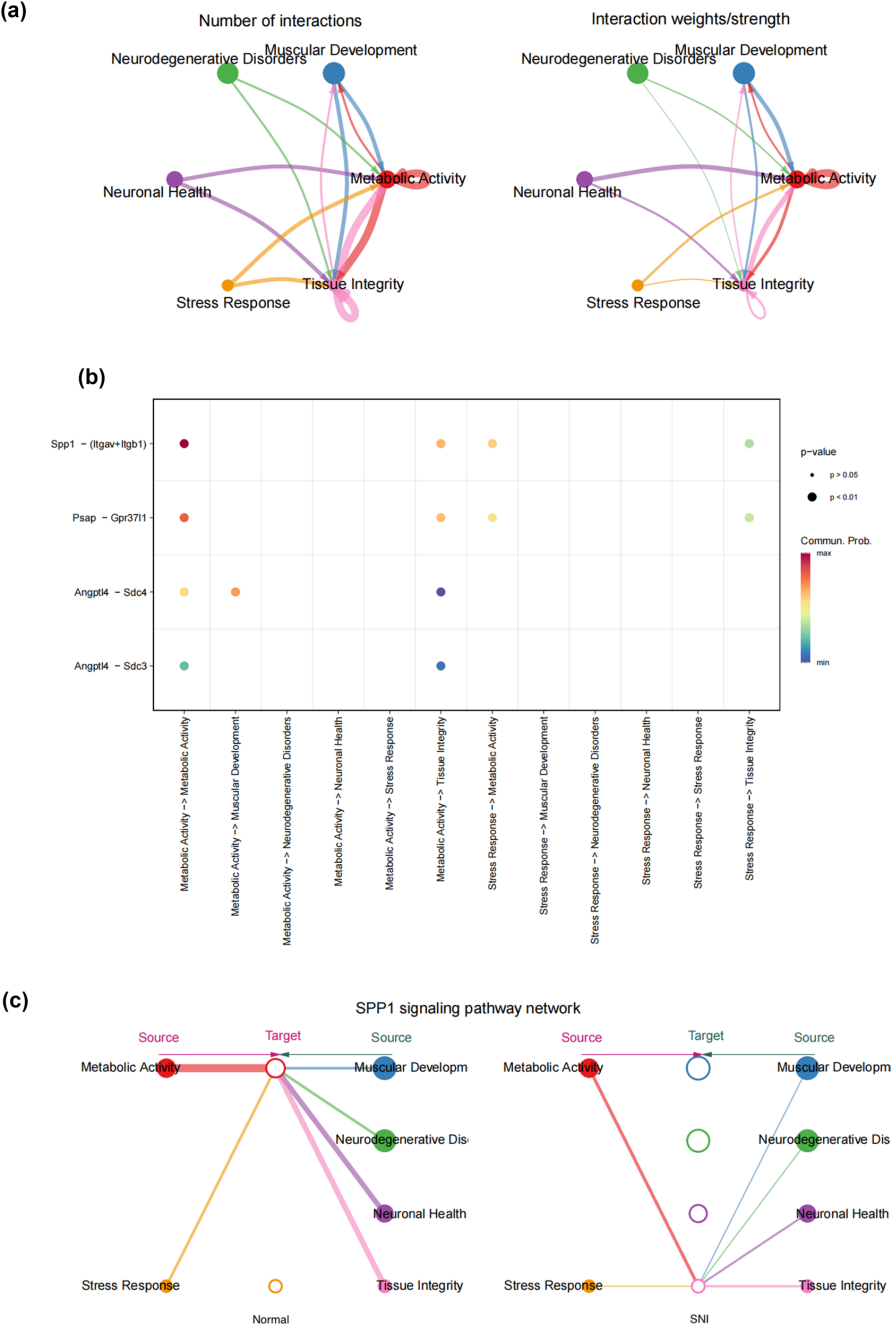
SPP1 signaling induces distinct SC interactions in DRG. (a) The number of interactions in a cell–cell communication network (the left panel); the interaction weights/strength in a cell–cell communication network (the left panel); (b) dot plots showing significant ligand–receptor pairs between different SC subtypes; and (c) overview of SPP1 signaling networks in six SC subtypes.

In this study, we employed the CellChat function to simulate the ligand–receptor interactions between different types of SCs in SNI, yielding a cell–cell communication network. More frequent cell–cell interactions between SRSCs, MASCs, and TISCs were observed (Figure 5a). Of note, SRSCs exhibited greater contact with MASCs via the SPP1 pathway (Figure 5b). Studies have shown that SPP1 is highly expressed in various models of neurological diseases, and it participates in different inflammatory responses by regulating immune cells [37,38,39,40,41]. In addition, SPPP1 signaling promotes the proliferation and survival of SCs after SNI [42]. We analyzed the intercellular signaling networks of SPP1, signaling to determine the important factors. SPP1 signaling is expressed paracrine in SRSCs. MASCs and TISCs are the main transmitters of the SPP1 signal (Figure 5c). The above results suggest that after the occurrence of SNI, the inflammatory environment SRSCs feel various stresses, followed by the interaction with MASCs and TISCs through SPP1 signaling, which promotes the two to perform neural repair functions. This shows that SPP1 signaling may become a promising therapeutic target for nerve injury repair.

Our findings suggest that SPP1 signaling could play a significant role in coordinating the response of SCs to peripheral nerve injury. By activating SCs, SPP1 signaling may enhance their ability to support axonal regeneration and remyelination, processes that are essential for effective nerve repair. Therefore, understanding the precise mechanisms by which SPP1 signaling influences SC behavior could provide valuable insights for developing novel therapeutic strategies aimed at enhancing peripheral nerve regeneration.

## 4 Discussion

Peripheral nerve injury, if not promptly and accurately repaired, can lead to permanent loss of peripheral nerve function. Current research shows that SCs participate in the clearance of debris, axonal and myelin regeneration, and re-innervation of target organs after peripheral nerve injury [43]. After peripheral nerve injury, SCs are rapidly activated and enter the repair process, undergoing a series of dynamic cellular remodeling changes, transforming into a repair phenotype, promoting nerve regeneration, guiding re-innervation of target organs, and thus restoring nerve function. There are many signaling pathways and transcriptional regulators that control these processes [7]. These repair functions after injury are typical functions of SCs in the peripheral nervous system [44,45]. However, Abdo et al. [46] have discovered a type of SCs with different repair functions: pain-sensitive SCs, which are distributed at the ends of pain-sensing neurons, forming a pain-sensing receptor organ network. It can be seen that there are still a large number of unknown, specific, functionally flexible, and specialized SCs in the entire organism.

Single-cell sequencing is beneficial for discovering cell types and subtypes and provides a more systematic and comprehensive understanding of cell fate [47,48]. In this study, we annotated and identified different cell subpopulations based on single-cell sequencing data of DRG retrieved from the GEO database. By analyzing the transcriptome of SCs in mouse DRG, we identified six SC subtypes with different biological functions. We mapped the pseudo-temporal states of SCs and inferred the differentiation order and progression of SC subtypes. Among these six types of cells, MASCs and TISCs with high metabolic activity in a proliferative state have the lowest degree of differentiation, have the potential to differentiate into other cell types, and may be the origin cells in the developmental trajectory of SCs, dedifferentiating to play a repair function after nerve injury. SC injury-induced dedifferentiation and subsequent nerve regeneration cell groups, while NDSCs are at the end and have no further development potential. In addition, we found a type of SCs different from the traditional repair function – SRSCs, which highly express *Npy* after injury. As a neuropeptide widely expressed in the nervous system, NPY plays a vital role in cortical excitability, stress response, food intake, circadian rhythm, and cardiovascular function [49,50,51,52,53]. In this study, GSEA results show that *Npy* is highly expressed in SRSCs after SNI, providing a potential theoretical basis for elucidating the regulatory role of SRSCs in stress response in SNI. At the same time, we found that SRSCs interact with other cells through the SPP1 signal, so we speculate that at the time of injury, SRSCs first sense the stimulus and send signals to other traditional repair cells through the SPP1 signaling pathway, prompting them to differentiate into progenitor cells to play a nerve repair function. Furthermore, we highlight the potential of SRSCs to serve as biomarkers indicative of the severity of neural injury and discuss the therapeutic significance of targeting SPP1 signaling. Understanding the role of SRSCs and their interaction with the SPP1 pathway could pave the way for developing novel therapeutic strategies aimed at enhancing neural repair. SPP1, through its interaction with its receptor on SCs, modulates cellular responses to injury by promoting the proliferation and migration of SCs, which are critical for the repair process. This signaling pathway may also contribute to the resolution of inflammation and the restoration of tissue homeostasis following nerve injury. Therefore, understanding the precise mechanisms by which SPP1 signaling influences SC behavior could provide valuable insights for developing novel therapeutic strategies aimed at enhancing peripheral nerve regeneration. Recent advancements in spatially resolved transcriptomics and proteomics offer new avenues for exploring cellular dynamics in response to injury. For instance, high-plex protein and whole transcriptome co-mapping at cellular resolution with spatial CITE-seq provide a comprehensive view of cellular responses [54]. Additionally, the spatial dynamics of mammalian brain development and neuroinflammation characterized by multimodal tri-omics mapping [55], and the spatially resolved *in vivo* CRISPR screen sequencing via perturb-DBiT [56], provide innovative approaches to study SC behavior in injury contexts. These techniques could offer future research avenues to explore the complex interactions between SRSCs, repair SCs, and nociceptive SCs and to further elucidate the role of SPP1 signaling in neural repair.

This study explores the cellular heterogeneity of SCs in peripheral nerve injury using single-cell sequencing technology, uncovering the distinct roles of SC subtypes in nerve repair. We identify abnormal expression patterns of key genes in specific subpopulations and emphasize the need for further research on the stimulus-sensing mechanisms of SRSCs to develop effective therapeutic strategies. The discovery of novel cell subtypes holds promise for treating peripheral nerve pain; however, further studies are required to evaluate their efficacy and safety in peripheral nerve disease treatment. While our research enhances the understanding of peripheral nerve disease pathogenesis and provides potential therapeutic targets, certain limitations remain. First, our study focuses exclusively on Scs, which may overlook the contributions of other cell types involved in nerve repair. Peripheral nerve regeneration is a complex process that involves not only Scs but also neurons, immune cells, and vascular components, all of which play essential roles in injury response and recovery. The absence of these cell types in our analysis may limit the scope of our findings. Therefore, future research should incorporate complementary single-cell datasets encompassing these additional cell types to provide a more comprehensive understanding of cellular interactions and regulatory networks in nerve regeneration. Additionally, the limited sample size and potential technical biases may affect the generalizability of our findings. Expanding the dataset and employing advanced computational approaches to correct for technical variation will be crucial for increasing the robustness of our conclusions. Finally, due to the lack of clinical samples, further validation is necessary to confirm our results and fully elucidate their underlying mechanisms. Future studies should adopt a more integrated and extensive approach, leveraging multimodal single-cell techniques and clinical validation, to deepen our understanding of peripheral nerve repair and facilitate the development of effective therapeutic strategies.

## Appendix

**Table A1:** Fold expression in different Schwann cell subtypes

	*p*_val	avg_log2FC	pct.1	pct.2	*p*_val_adj	Cluster	Gene
Thy1	1.56 × 10^−8^	0.441326	0.531	0.514	0.000402	Stress response	THY1
Plp1	2.80 × 10^−76^	0.422452	0.957	0.873	7.21 × 10^−72^	Muscular development	PLP1
Gap43	2.45 × 10^−52^	0.537436	0.544	0.385	6.31 × 10^−48^	Muscular development	GAP43
S100a6	6.14 × 10^−30^	0.317475	0.993	0.986	1.58 × 10^−25^	Stress response	S100A6

## References

[1] Wang ML, Rivlin M, Graham JG, Beredjiklian PK. Peripheral nerve injury, scarring, and recovery. Connect Tissue Res. 2019;60(1):3–9. 10.1080/03008207.2018.1489381 30187777

[2] Li NY, Onor GI, Lemme NJ, Gil JA. Epidemiology of peripheral nerve injuries in sports, exercise, and recreation in the United States, 2009 - 2018. Phys Sportsmed. 2021;49(3):355–62. 10.1080/00913847.2020.1850151 33187455

[3] Modrak M, Talukder MH, Gurgenashvili K, Noble M, Elfar JC. Peripheral nerve injury and myelination: Potential therapeutic strategies. J Neurosci Res. 2020;98(5):780–95. 10.1002/jnr.24538 PMC7072007 31608497

[4] Zhang M, Li L, An H, Zhang P, Liu P. Repair of peripheral nerve injury using hydrogels based on self-assembled peptides. Gels. 2021;7(4):152. 10.3390/gels7040152 PMC8544532 34698159

[5] Bolívar S, Navarro X, Udina E. Schwann cell role in selectivity of nerve regeneration. Cells. 2020;9(9):2131. 10.3390/cells9092131 PMC7563640 32962230

[6] Jessen KR, Mirsky R, Lloyd AC. Schwann cells: Development and role in nerve repair. Cold Spring Harb Perspect Biol. 2015;7(7):a020487. 10.1101/cshperspect.a020487 PMC4484967 25957303

[7] Nocera G, Jacob C. Mechanisms of Schwann cell plasticity involved in peripheral nerve repair after injury. Cell Mol Life Sci. 2020;77(20):3977–89. 10.1007/s00018-020-03516-9 PMC7532964 32277262

[8] Bellamkonda RV. Peripheral nerve regeneration: an opinion on channels, scaffolds and anisotropy. Biomaterials. 2006;27(19):3515–8. 10.1016/j.biomaterials.2006.02.030 16533522

[9] Dai LG, Huang GS, Hsu SH. Sciatic nerve regeneration by cocultured Schwann cells and stem cells on microporous nerve conduits. Cell Transplant. 2013;22(11):2029–39. 10.3727/096368912X658953 23192007

[10] Jessen KR, Mirsky R. The origin and development of glial cells in peripheral nerves. Nat Rev Neurosci. 2005;6(9):671–82. 10.1038/nrn1746 16136171

[11] Scheib J, Höke A. Advances in peripheral nerve regeneration. Nat Rev Neurol. 2013;9(12):668–76. 10.1038/nrneurol.2013.227 24217518

[12] Gerber D, Pereira JA, Gerber J, Tan G, Dimitrieva S, Yángüez E, et al. Transcriptional profiling of mouse peripheral nerves to the single-cell level to build a sciatic nerve ATlas (SNAT). Elife. 2021;10:e58591. 10.7554/eLife.58591 PMC8064760 33890853

[13] Kastriti ME, Faure L, Von Ahsen D, Bouderlique TG, Boström J, Solovieva T, et al. Schwann cell precursors represent a neural crest-like state with biased multipotency. Embo J. 2022;41(17):e108780. 10.15252/embj.2021108780 PMC9434083 35815410

[14] Wolbert J, Li X, Heming M, Mausberg AK, Akkermann D, Frydrychowicz C, et al. Redefining the heterogeneity of peripheral nerve cells in health and autoimmunity. Proc Natl Acad Sci U S A. 2020;117(17):9466–76. 10.1073/pnas.1912139117 PMC7196786 32295886

[15] Yim AK, Wang PL, Bermingham Jr JR, Hackett A, Strickland A, Miller TM, et al. Disentangling glial diversity in peripheral nerves at single-nuclei resolution. Nat Neurosci. 2022;25(2):238–51. 10.1038/s41593-021-01005-1 PMC9060899 35115729

[16] Stuart T, Butler A, Hoffman P, Hafemeister C, Papalexi E, Mauck WM, et al. Comprehensive integration of single-cell data. Cell. 2019;177(7):1888–902. 10.1016/j.cell.2019.05.031 PMC6687398 31178118

[17] Korsunsky I, Millard N, Fan J, Slowikowski K, Zhang F, Wei K, et al. Fast, sensitive and accurate integration of single-cell data with harmony. Nat Methods. 2019;16(12):1289–96. 10.1038/s41592-019-0619-0 PMC6884693 31740819

[18] Aran D, Looney AP, Liu L, Wu E, Fong V, Hsu A, et al. Reference-based analysis of lung single-cell sequencing reveals a transitional profibrotic macrophage. Nat Immunol. 2019;20(2):163–72. 10.1038/s41590-018-0276-y PMC6340744 30643263

[19] Sherman BT, Hao M, Qiu J, Jiao X, Baseler MW, Lane HC, et al. DAVID: a web server for functional enrichment analysis and functional annotation of gene lists (2021 update). Nucleic Acids Res. 2022;50(W1):W216–w21. 10.1093/nar/gkac194 PMC9252805 35325185

[20] Zhou Y, Zhou B, Pache L, Chang M, Khodabakhshi AH, Tanaseichuk O, et al. Metascape provides a biologist-oriented resource for the analysis of systems-level datasets. Nat Commun. 2019;10(1):1523. 10.1038/s41467-019-09234-6 PMC6447622 30944313

[21] Jin S, Guerrero-Juarez CF, Zhang L, Chang I, Ramos R, Kuan CH, et al. Inference and analysis of cell-cell communication using CellChat. Nat Commun. 2021;12(1):1088. 10.1038/s41467-021-21246-9 PMC7889871 33597522

[22] Haghverdi L, Büttner M, Wolf FA, Buettner F, Theis FJ. Diffusion pseudotime robustly reconstructs lineage branching. Nat Methods. 2016;13(10):845–8. 10.1038/nmeth.3971 27571553

[23] Trapnell C, Cacchiarelli D, Grimsby J, Pokharel P, Li S, Morse M, et al. The dynamics and regulators of cell fate decisions are revealed by pseudotemporal ordering of single cells. Nat Biotechnol. 2014;32(4):381–6. 10.1038/nbt.2859 PMC4122333 24658644

[24] Niehaus JK, Taylor-Blake B, Loo L, Simon JM, Zylka MJ. Spinal macrophages resolve nociceptive hypersensitivity after peripheral injury. Neuron. 2021;109(8):1274–82. 10.1016/j.neuron.2021.02.018 PMC8068642 33667343

[25] Wang K, Wang S, Chen Y, Wu D, Hu X, Lu Y, et al. Single-cell transcriptomic analysis of somatosensory neurons uncovers temporal development of neuropathic pain. Cell Res. 2021;31(8):904–18. 10.1038/s41422-021-00479-9 PMC8324866 33692491

[26] Babetto E, Wong KM, Beirowski B. A glycolytic shift in Schwann cells supports injured axons. Nat Neurosci. 2020;23(10):1215–28. 10.1038/s41593-020-0689-4 PMC8758250 32807950

[27] Pestronk A, Schmidt RE, Bucelli R, Sim J. Schwann cells and myelin in human peripheral nerve: Major protein components vary with age, axon size and pathology. Neuropathol Appl Neurobiol. 2023;49(2):e12898. 10.1111/nan.12898 36868780

[28] Ramli K, Aminath Gasim I, Ahmad AA, Hassan S, Law ZK, Tan GC, et al. Human bone marrow-derived MSCs spontaneously express specific Schwann cell markers. Cell Biol Int. 2019;43(3):233–52. 10.1002/cbin.11067 30362196

[29] Shackleford GG, Marziali LN, Sasaki Y, Claessens A, Ferri C, Weinstock NI, et al. A new mouse model of Charcot-Marie-Tooth 2J neuropathy replicates human axonopathy and suggest alteration in axo-glia communication. PLoS Genet. 2022;18(11):e1010477. 10.1371/journal.pgen.1010477 PMC9707796 36350884

[30] Thomaidou D, Coquillat D, Meintanis S, Noda M, Rougon G, Matsas R. Soluble forms of NCAM and F3 neuronal cell adhesion molecules promote Schwann cell migration: identification of protein tyrosine phosphatases zeta/beta as the putative F3 receptors on Schwann cells. J Neurochem. 2001;78(4):767–78. 10.1046/j.1471-4159.2001.00454.x 11520897

[31] Woods C, Kapur RP, Bischoff A, Lovell M, Arnold M, Peña A, et al. Neurons populating the rectal extrinsic nerves in humans express neuronal and Schwann cell markers. Neurogastroenterol Motil. 2021;33(7):e14074. 10.1111/nmo.14074 33382200

[32] Ceci FM, Ferraguti G, Petrella C, Greco A, Tirassa P, Iannitelli A, et al. Nerve growth factor, stress and diseases. Curr Med Chem. 2021;28(15):2943–59. 10.2174/0929867327999200818111654 32811396

[33] Costa LS, Aidar FJ, Matos DGD, Oliveira JUD, Santos JLD, Almeida-Neto PFD, et al. Effects of resistance training and Bowdichia virgilioides Hydroethanolic extract on oxidative stress markers in rats submitted to peripheral nerve injury. Antioxidants. 2020;9(10):941. 10.3390/antiox9100941 PMC7601135 33019503

[34] Qian Y, Han Q, Zhao X, Song J, Cheng Y, Fang Z, et al. 3D melatonin nerve scaffold reduces oxidative stress and inflammation and increases autophagy in peripheral nerve regeneration. J Pineal Res. 2018;65(4):e12516. 10.1111/jpi.12516 29935084

[35] Cheng YC, Chu LW, Chen JY, Hsieh SL, Chang YC, Dai ZK, et al. Loganin attenuates high glucose-induced schwann cells pyroptosis by inhibiting ROS generation and NLRP3 inflammasome activation. Cells. 2020;9(9):1948. 10.3390/cells9091948 PMC7564733 32842536

[36] Li Z, Wu F, Xu D, Zhi Z, Xu G. Inhibition of TREM1 reduces inflammation and oxidative stress after spinal cord injury (SCI) associated with HO-1 expressions. Biomed Pharmacother. 2019;109:2014–21. 10.1016/j.biopha.2018.08.159 30551457

[37] Adlerberth ANNIKA, Stenström G, Hasselgren PO. The selective beta 1-blocking agent metoprolol compared with antithyroid drug and thyroxine as preoperative treatment of patients with hyperthyroidism. Results from a prospective, randomized study. Ann Surg. 1987;205(2):182–8. 10.1097/00000658-198702000-00013 PMC1492817 3545108

[38] Argandona Lopez C, Brown AM. Microglial-neuronal crosstalk in chronic viral infection through mTOR, SPP1/OPN and inflammasome pathway signaling. Front Immunol. 2024;15:1368465. 10.3389/fimmu.2024.1368465 PMC11032048 38646526

[39] De Schepper S, Ge JZ, Crowley G, Ferreira LS, Garceau D, Toomey CE, et al. Perivascular cells induce microglial phagocytic states and synaptic engulfment via SPP1 in mouse models of Alzheimer’s disease. Nat Neurosci. 2023;26(3):406–15. 10.1038/s41593-023-01257-z PMC9991912 36747024

[40] Lan Y, Zhang X, Liu S, Guo C, Jin Y, Li H, et al. Fate mapping of Spp1 expression reveals age-dependent plasticity of disease-associated microglia-like cells after brain injury. Immunity. 2024;57(2):349–63. 10.1016/j.immuni.2024.01.008 38309272

[41] Lopes KDP, Yu L, Shen X, Qiu Y, Tasaki S, Iatrou A, et al. Associations of cortical SPP1 and ITGAX with cognition and common neuropathologies in older adults. Alzheimers Dement. 2024;20(1):525–37. 10.1002/alz.13474 PMC10841499 37727065

[42] Wang JB, Zhang Z, Li JN, Yang T, Du S, Cao RJ, et al. SPP1 promotes Schwann cell proliferation and survival through PKCα by binding with CD44 and αvβ3 after peripheral nerve injury. Cell Biosci. 2020;10:98. 10.1186/s13578-020-00458-4 PMC7439540 32843960

[43] Bosch-Queralt M, Fledrich R, Stassart RM. Schwann cell functions in peripheral nerve development and repair. Neurobiol Dis. 2023;176:105952. 10.1016/j.nbd.2022.105952 36493976

[44] Jessen KR, Mirsky R. The repair Schwann cell and its function in regenerating nerves. J Physiol. 2016;594(13):3521–31. 10.1113/JP270874 PMC4929314 26864683

[45] Jessen KR, Mirsky R. The success and failure of the Schwann cell response to nerve injury. Front Cell Neurosci. 2019;13:33. 10.3389/fncel.2019.00033 PMC6378273 30804758

[46] Abdo H, Calvo-Enrique L, Lopez JM, Song J, Zhang MD, Usoskin D, et al. Specialized cutaneous Schwann cells initiate pain sensation. Science. 2019;365(6454):695–9. 10.1126/science.aax6452 31416963

[47] Bi Y, Jing Y, Guo L. Construction and validation of a prognostic marker and risk model for HCC ultrasound therapy combined with WGCNA identification. Front Genet. 2022;13:1017551. 10.3389/fgene.2022.1017551 PMC9573990 36263426

[48] Peng S, Hebert LL, Eschbacher JM, Kim S. Single-Cell RNA sequencing of a postmenopausal normal breast tissue identifies multiple cell types that contribute to breast cancer. Cancers. 2020;12(12):3639. 10.3390/cancers12123639 PMC7761899 33291647

[49] Félétou M, Galizzi JP, Levens NR. NPY receptors as drug targets for the central regulation of body weight. CNS Neurol Disord Drug Targets. 2006;5(3):263–74. 10.2174/187152706777452236 16787228

[50] Groneberg DA, Folkerts G, Peiser C, Chung KF, Fischer A. Neuropeptide Y (NPY). Pulm Pharmacol Ther. 2004;17(4):173–80. 10.1016/j.pupt.2004.04.003 15219262

[51] Kohno D, Yada T. Arcuate NPY neurons sense and integrate peripheral metabolic signals to control feeding. Neuropeptides. 2012;46(6):315–9. 10.1016/j.npep.2012.09.004 23107365

[52] Mercer RE, Chee MJ, Colmers WF. The role of NPY in hypothalamic mediated food intake. Front Neuroendocrinol. 2011;32(4):398–415. 10.1016/j.yfrne.2011.06.001 21726573

[53] Schmeltzer SN, Herman JP, Sah R. Neuropeptide Y (NPY) and posttraumatic stress disorder (PTSD): A translational update. Exp Neurol. 2016;284(Pt B):196–210. 10.1016/j.expneurol.2016.06.020 PMC8375392 27377319

[54] Liu Y, DiStasio M, Su G, Asashima H, Enninful A, Qin X, et al. High-plex protein and whole transcriptome co-mapping at cellular resolution with spatial CITE-seq. Nat Biotechnol. 2023;41(10):1405–9. 10.1038/s41587-023-01676-0 PMC10567548 36823353

[55] Fan R, Zhang D, Rodríguez-Kirby L, Lin Y, Song M, Wang L, et al. Spatial dynamics of mammalian brain development and neuroinflammation by multimodal tri-omics mapping. bioRxiv. 2024;605493.

[56] Baysoy A, Tian X, Zhang F, Renauer P, Bai Z, Shi H, et al. Spatially resolved in vivo CRISPR screen sequencing via Perturb-DBiT. bioRxiv. 2024;624106.

